# SBAR-LA: SBAR Brief Assessment Rubric for Learner Assessment

**DOI:** 10.15766/mep_2374-8265.11184

**Published:** 2021-10-18

**Authors:** Beth P. Davis, Sally A. Mitchell, Jeannie Weston, Catherine Dragon, Munish Luthra, James Kim, Hugh A. Stoddard, Douglas S. Ander

**Affiliations:** 1 Associate Professor, Division of Physical Therapy, Department of Rehabilitation Medicine, Emory University School of Medicine; 2 Assistant Professor of Clinical Anesthesia, Vice Chair of Education, and Statewide Assistant Clerkship Director, Department of Anesthesia, Indiana University School of Medicine; 3 Assistant Professor, Emory University School of Nursing; 4 Assistant Professor, Division of Physician Assistant, Department of Family and Preventive Medicine, Emory University School of Medicine; 5 Assistant Professor, Department of Medicine, Emory University School of Medicine; 6 Associate Professor, Department of Medicine, Emory University School of Medicine; 7 Professor, Department of Medicine, Emory University School of Medicine; 8 Professor, Department of Emergency Medicine, Emory University School of Medicine

**Keywords:** Editor's Choice, Interprofessional Education, Interprofessional Communication, SBAR, Communication Assessment, Case-Based Learning, Program Evaluation, Team-Based Learning

## Abstract

**Introduction:**

Structured communication tools are associated with improvement in information transfer and lead to improved patient safety. Situation, Background, Assessment, Recommendation (SBAR) is one such tool. Because there is a paucity of instruments to measure SBAR effectiveness, we developed and validated an assessment tool for use with prepractice health professions students.

**Methods:**

We developed the SBAR Brief Assessment Rubric for Learner Assessment (SBAR-LA) by starting with a preliminary list of items based on the SBAR framework. During an interprofessional team training event, students were trained in the use of SBAR. Subsequently, they were assigned to perform a simulated communication scenario demonstrating use of SBAR principles. We used 10 videos from these scenarios to refine the items and scales over two rounds. Finally, we applied the instrument on another subset of 10 students to conduct rater calibration and measure interrater reliability.

**Results:**

We used a total of 20 out of 225 videos of student performance to create the 10-item instrument. Interrater reliability was .672, and for eight items, the Fleiss’ kappa was considered good or fair.

**Discussion:**

We developed a scoring rubric for teaching SBAR communication that met criteria for validity and demonstrated adequate interrater reliability. Our development process provided evidence of validity for the content, construct, and response process used. Additional evidence from the use of SBAR-LA in settings where communication skills can be directly observed, such as simulation and clinical environments, may further enhance the instrument's accuracy. The SBAR-LA is a valid and reliable instrument to assess student performance.

## Educational Objectives

By using this assessment, facilitators will be able to:

1. Assess student skill in applying the Situation, Background, Assessment, Recommendation (SBAR) framework for communication with other health care professionals.
2. Demonstrate reliability in the use of the instrument to assess student communication skills.
3. Use the data to inform the evaluation of the efficacy of interprofessional education activities.

## Introduction

The 1999 Institute of Medicine report *To Err Is Human* sparked national attention and a prioritization of health care quality and patient safety initiatives.^1^ Communication failures have been estimated to be a major factor in 60%-70% of serious health care-related accidents.^1–4^ During the transfer of information between providers, inadequate communication of vital information can occur, resulting in medical errors that can be fatal.^3,5–8^ Standardized communication is one means of improving safety during this transfer of information. While it has been demonstrated that structured communication has benefit, outcomes-based literature needs to be more robust.^9–11^ For this study, prelicensure health care professionals from six disciplines used a structured communication tool (i.e., checklist). This tool was associated with an improvement in the transfer of information and reduction in omission of information expected on the part of the recipient.^12–14^ One structure suggested to improve communication was the situational briefing tool, Situation, Background, Assessment, Recommendation (SBAR).^15^ The Institute for Healthcare Improvement has listed SBAR as the model health providers should use to structure clinical communication.^16,17^

SBAR has been noted to be an effective structured clinical communication tool and is a component of the TeamSTEPPS curriculum.^18^ A literature search included PubMed, CINAHL, Embase, and Google Scholar. Keywords included SBAR, communication, and assessment. One article demonstrated higher SBAR communication skills after participating in an educational program for senior-year nursing students.^19^ In that study, the researchers used a checklist developed by Mi Yu and Kyung ja Kang^20^ that contained 12 items in total to measure each of the SBAR subscales. A 3-point scale (0 = *no performance*, 1 = *lacking*, 2 = *reasonable*) was used to assess global effectiveness.^20^ Internal consistency, as measured by Cronbach's alpha, was .58.^20^ No further validity evidence or conformational studies for this scale were found in the literature. Another study measured the effectiveness of using SBAR among first-year medical students in a group simulation scenario. Raters used a global effectiveness rating on a 5-point scale and a 20-point list for SBAR items mentioned by the students.^21^ Authors from this study reported reliability with kappa values of .55 to 1.0 between two independent coders who observed 10 of the 17 groups.^21^ For the global effectiveness rating scale, authors reported an absolute agreement between the two observers (*K* = 1.0, *p* < .001).^21^ A third paper focused on SBAR training of nurses in a patient fall scenario.^22^ This study utilized a nine-item tool based on previous work by Jennifer Dunsford.^22,23^ Tool validity was established by professors in nursing and simulation.^22^ Reliability was established through review by an external expert, and they attained a Cronbach's alpha of .78.^22^

We had begun this interprofessional education (IPE) activity with the goal of designing and delivering an effective educational intervention to train learners to use SBAR; however, the paucity of valid learner assessment tools for the intervention precluded quantitative evaluation of the learning outcomes from the activity. Although several tools have been noted in the literature, we found that they lacked sufficient validity evidence or measured reliability. Thus, we set out to create and critique an instrument using Messick's validity criteria,^24^ titled the SBAR Brief Assessment Rubric for Learner Assessment (SBAR-LA), for assessing learner performance in an IPE activity that was based on the SBAR interprofessional communication framework. We describe the design and development of an assessment tool for measuring learner achievement at employing the SBAR framework for communication in a simulation environment.

## Methods

### Educational Activity

The committee for Interprofessional Team Training Day (ITTD) created a curriculum at our academic health center in 2007. Each year since, the committee has revised and expanded the program, which currently includes over 1,200 students from eight health professions education programs in the schools of medicine, nursing, and public health. The ITTD curriculum addressed each of the four Interprofessional Education Collaborative core competencies.^25^ Students from the schools of medicine and nursing attend two ITTD sessions during their educational program. Students from the school of public health attended only the first ITTD session and were not included in this study.

The ITTD event began with a large-group lecture for all students to explain the importance of teamwork, communication, and roles within a team. Additionally, the lecture and panel discussion highlighted the importance of values and ethics for establishing and maintaining a climate of mutual respect and shared values in teamwork. Students then divided into small, interprofessional groups with a trained faculty facilitator. The focus of the small-group case study was interprofessional communication. The facilitator taught the skills for exchange of information between health care providers using SBAR. Students were given examples of how to use SBAR, and then they practiced the skills with case studies. Each student practiced being the sender and receiver of information using SBAR.

In addition to the ITTD activities, students were assigned to perform a simulated SBAR communication scenario twice, once before and once after the ITTD instructional sessions. For this assignment, students were provided with a written clinical vignette and directed to use SBAR to communicate critical health care information. They made audiovisual recordings of themselves simulating this communication (Appendix A). The instructions were to “simply record your response to the best of your ability with respect to your prior knowledge and background experience” using the audiovisual recording feature in Canvas Learning Management System (Instructure). The students received automated email notifications with instructions for using Canvas to complete the assignments. The students received the notification to submit the precourse recording 5 days in advance, and the postcourse recording was due 24 hours after ITTD. The data collection and study design were determined to be exempt from review by our university's institutional review board (IRB00091030).

### Instrument Development

The SBAR scoring rubric was devised by the authors of this study, all of whom were members of the ITTD planning group. The study team contained members from health care professions participating in ITTD, all of whom had more than 5 years of experience with interprofessional training at the time, and all were experienced health care providers and educators within their disciplines: anesthesiologist assistant (one member), medicine (three members), nursing (one member), physical therapy (one member), and physician assistant (one member). Each member of the study team also served as a rater; therefore, their expertise regarding SBAR as a communication tool and the scoring rubric was obtained through years of experience teaching the course and involvement in the research team. All members of the research team/raters were from our home institution at the time of the study. An expert, nonclinician medical education researcher assisted the team with the instrument creation, study design, and data analysis.

The initial outline of the scoring rubric was designed by an expert panel composed of the ITTD team members described above. The four categories of the SBAR framework were used to define the constructs that would be measured.^15,16,24–28^ The content validity of the instrument was predicated on operationalizing the critical communications statements that should be expected of prepractice health professions students. These communications statements constituted the content measured by SBAR-LA. The team initially operationalized SBAR constructs into 15 subcategories. Each of these subcategories aligned with a specific statement of information by the student that the expert panel deemed to be necessary for the effective use of SBAR in practice. A global effectiveness rating (GER) item was added to the rubric exclusively for the purpose of estimating the internal consistency of the tool. Adding the GER allowed for comparison between the analytic measurements from the 15 subcategories and the global measurement of the GER. Such a comparison was included to provide evidence that students’ scores were genuine and were not an artifice of the scoring method used. While creating the rubric, the authors used some pre- and postevent audiovisual recordings as a reference for developers to discuss and compare perspectives on the tool's construct representation and the levels of student performance.

### Response Process Validity and Pilot Testing

The rubric was intended for use by expert raters to observe the student audiovisuals and assign a score based on learner performance in each of the initial 15 subcategories. Based on demonstration of appropriate SBAR statements by the student, the score range for each subcategory was rated on a 3-point scale (0 = *unsuccessful*, 1 = *attempted*, 2 = *successful*). Similarly, the GER scale used a 3-point scale (0 = *not effective*, 1 = *moderately effective*, 2 = *very effective*). The points for each of the 15 subcategories were summed to create a composite score that would represent the rater's assessment of the construct communication as defined by the SBAR framework (Appendix B).

The authors randomly selected five student audiovisual recording submissions to internally hone and calibrate the rubric of 15 items. The five clips were selected from the pool of 225 subjects who had submitted both a pre- and postevent recording. In a group setting, each rater independently scored each of the five clips. A group discussion ensued during which raters compared points awarded to each subject for each item along with the rationale and justifications for their individual scoring. These discussions pinpointed subcategories that were operationally redundant and others for which the descriptions needed greater specificity. Based on these discussions, the authors edited and deleted subcategories and descriptions to more precisely measure student communication using SBAR. After three rounds of this scoring and discussion process, there was a convergence of raters’ judgments. Subsequently, the authors independently scored a new group of five audiovisual recording submissions, and there was a reevaluation of how well scoring between the authors converged. The authors made further editorial adjustments to the draft rubric until all the raters agreed on a final version. This final SBAR rubric reduced the number of subcategories to 10, which were discrete, clearly defined, and readily detectible from the audiovisual recordings. These 10 subcategories included four statements related to situation, three related to background, one to assessment, and two for recommendation. Each subcategory had a maximum score of 2, yielding a best possible score of 20 points. The GER score had a maximum of 2 points; however, the authors did not sum the 10 analytical, subcategory scores with the global GER score (Appendix B).

### Rater Calibration

To verify the utility of the process and instrument, seven authors (Beth P. Davis, Sally A. Mitchell, Jeannie Weston, Catherine Dragon, James Kim, Munish Luthra, and Douglas S. Ander) were assigned to score 10 previously unseen SBAR audiovisual recordings using the rubric. For this first round, each audiovisual recording was scored independently by the raters. During a follow-up group discussion, scoring discrepancies were discussed to determine the root source of any remaining disagreements. The raters found the scoring system easy to apply and the subcategories to be sufficiently discrete, and they sensed that the total score (i.e., the sum of the subcategory scores) was consistent with their holistic perception of the students’ overall performance. Nonetheless, to promote rater consistency—particularly in consideration of eventual use by raters not involved in the development and validation of the rubric—several of the score descriptions for subcategories were edited for clarity and precision.

The scores assigned during the final rater-calibration exercise were used to calculate interrater reliability using the SBAR-LA rubric. Following the rater training and instrument revision noted above, scores from 10 randomly selected students were used for reliability estimation. Scores for each student at both pre- and postevent recording were analyzed using Krippendorff's alpha for interval scores.^29^ A reliability estimate was calculated for each of the 10 items individually and for the total score (i.e., the sum of the 10 items). A separate reliability estimate for the single global item was also made. Although the reliability of the global item was substandard, the global rating correlated strongly with the analytic total score; the consistency between these two approaches supported overall consistency of the instrument.

### Reliability Estimate

The initial reliability estimates for the 10 individual items, the global item, and the total score yielded borderline values; however, transforming each item score into a dichotomous variable (0, 1) yielded much stronger reliability statistics. This transformation to dichotomous scoring was necessary to account for raters’ uncertainty about the difference between a score of 1 or 2. Raters appeared to be tentative about judging the quality of student performance that would lead to differentiation between partial completion (score of 1) and full completion (score of 2) for a task. On the other hand, there was no uncertainty as to whether a student had at least attempted a task or not, which was captured by the dichotomous scoring. This rater phenomenon led to a significant modification to our initial scoring plan. Now, each item is scored dichotomously (0 or 1), leading to a total score with a maximum of 10 points. This approach is also more consistent with a criterion-referenced scoring approach (as opposed to a norm-referenced scoring approach, which would maximize discrimination between examinees). The final rubric with categories, subcategories, and descriptions is presented in Appendix B.

## Results

Using the dichotomous scoring of items, the Krippendorff's alpha for the total score of the 10 items was .672. This result was above the minimum level for instrument usefulness, albeit lower than ideal.^29^ The reliability of the 10 individual, dichotomously scored items was calculated using Fleiss’ kappa for categorical data with multiple raters.^30^ For eight of the 10 items, the Fleiss’ kappa was good or fair; two items had weak reliability, which will need to be addressed through rater training prior to subsequent use of the SBAR-LA.

## Discussion

Effective communication is paramount in the health care field for reducing medical errors and improving quality of care. Training health professions students in effective communication skills is an important part of their preparation for clinical practice, and that importance is evident from the literature. SBAR is a tool utilized in many health care settings for structured and clear communication. SBAR, as we have noted, is a simple structured communication tool that can be used by all providers at varying levels of experience. However, the ability to assess whether SBAR training is adequate for effective communication is limited. This gap in our ability to assess SBAR performance led us to design and develop the SBAR-LA. Our aim was to create a valid and reliable assessment scoring rubric to primarily measure (1) the effectiveness of an SBAR training module and (2) the ability of any learner to effectively use this communication tool.

Our work to develop a valid scoring tool to assess SBAR in a simulated, interprofessional environment is unique. Previous work demonstrated that SBAR could be assessed^21^; however, the SBAR-LA is the first scoring rubric for preprofessional learners. We were able to demonstrate adequate interrater reliability, and our development process was conducted to generate sound evidence for the validity of the content, construct, and response process. For the future, reliability will be recalculated for use of the instrument by fewer raters, which is a more likely scenario than the seven concurrent raters used for the pilot testing. Similarly, revisions to the wording of the items to aid in rater consistency should improve reliability. Such modifications will be guided using generalizability theory approaches to reliability calculations.

In a review article on the use of scoring rubrics, it was noted that the ability to provide valid and reliable scoring could also be leveraged as a tool for learning.^26,27^ For example, SBAR-LA could be used both as an assessment instrument and as a tool for supplying specific, formative feedback to examinees. While creating our rubric, the inclusion and refinement of descriptive anchors allowed for more reliable assessment of the learners’ SBAR skills. The same descriptors can be used for teaching purposes by providing the faculty and learner with clear descriptions for each component of SBAR. These descriptions align with the teaching of SBAR and can be easily aligned with the feedback that is provided to learners following the assessment of their skills.

While the scenario featured a nurse giving a report to a physician, the exercise was not limited to nursing students reporting to medical students. All of the students, regardless of their educational track, were asked to craft a verbal report from the perspective of the on-shift health care provider (e.g., health care student, nurse, advanced practice provider, resident physician) conveying concern to the patient's primary provider (e.g., physician, advanced practice provider, chief resident, intern) about a change in the patient's condition. The patient demonstrated a change in mental status after a fall that could represent an intracranial bleed since the patient was on the blood thinner Coumadin. This should prompt the nurse to call the other provider with the recommendation of further evaluation by bedside examination and/or expediting the planned CT scan.

This communication skill is transferable regardless of what professional role the student will have upon graduation. The usefulness of this tool derives from the relative uniformity and efficiency through which critical information can be conveyed. This technique can be used between members of the same professional group (e.g., physicians communicating with other physicians) and also for interprofessional communication between providers (physical therapist and nurse, nurse and anesthesiologist assistant, physician assistant and physician, etc.). The exercise is also useful for interprofessional team building in that it allows for non-nursing students to imagine the type of role a nurse would play in this situation. While we selected a nurse-provider communication scenario, other scenarios involving different health care or public health providers could be used. Appropriate medical information was provided to all students regardless of discipline; in this way, students from all disciplines were able to deliver critical information.

### Limitations

The rubric was designed to measure performance of SBAR communication in a simulated educational environment. Also, the case was provided in a written format and recorded in a nonstressful setting. The use of this tool to measure performance in a clinical environment was not assessed and would certainly require additional evidence for validation in that context. Also, although the interprofessional training session that was used for development of this rubric included multiple health profession training programs, additional evidence may be necessary before extending SBAR-LA use to other health care professions training programs, such as those that are not directly involved in patient handoffs.

### Conclusion

A scoring rubric was developed to align with teaching the SBAR communication framework to prepractice health professions students. This rubric, SBAR-LA, demonstrated robust validity and adequate reliability. The instrument has been shown here to be capable of measuring learner performance in employing SBAR to communicate information in a simulated, interprofessional context. For the future, SBAR-LA can be employed by health professions education programs to accurately measure the effectiveness of SBAR training events, such as ITTD, as it relates to IPE and communication. Having a simple, valid tool for measuring student learning in IPE fills an oft-noted gap and provides the groundwork for creating, evaluating, and improving upon IPE instruction for prepractice students.

## Appendices


Student SBAR Assignment.docxSBAR-LA Rubric.docx
*All appendices are peer reviewed as integral parts of the Original Publication.*
