# Supplementary material for: SBAR-LA: SBAR Brief Assessment Rubric for Learner Assessment
Source: MedEdPORTAL. 2021 Oct 18;17:11184. doi: 10.15766/mep_2374-8265.11184 (PMC8520891; doi:10.15766/mep_2374-8265.11184)
Supplement: Supplementary file 1 — Student SBAR Assignment.docxSBAR-LA Rubric.docx [file mep_2374-8265.11184-s001.zip › A. Student SBAR Assignment.docx]

APPENDIX A. Student SBAR Assignment

Use the Canvas media recording option to make a video of your response to the provided clinical scenario and prompts.  Find a quiet environment with minimal background noise.  To properly analyze the responses, instructors will need clear, audible recordings.  This activity is best done on a laptop or desktop computer with a camera and microphone.

Case Scenario: Joan Willis, DOB 4-4-54, a female was brought to the hospital after “falling at home.” Ms. Willis sustained a right radius/ulnar fracture, bruising, a laceration to her head, and brief loss of consciousness. She was admitted to the medical surgical observation unit overnight. Her orders include hourly neurovascular checks and a repeat CT scan ordered for “in the morning.” Her son is also bringing in her medication list from home. During her last neurovascular check, she has become somewhat confused, a change from the previous hour’s assessment. Her son has arrived with her medication list. One of the medications that she regularly takes is Warfarin 5 mg/day.

Exercise: You are the nurse for Ms. Willis, and you want to call her physician to report her recent change in assessment along with other pertinent information. She has a CT scan scheduled for “in the morning” and it is now 2AM. You feel the change in her assessment may be due to a possible intracranial hemorrhage due to the fall and her daily use of Warfarin. Verbally communicate these findings to the physician.
