# Supplementary material for: SBAR-LA: SBAR Brief Assessment Rubric for Learner Assessment
Source: MedEdPORTAL. 2021 Oct 18;17:11184. doi: 10.15766/mep_2374-8265.11184 (PMC8520891; doi:10.15766/mep_2374-8265.11184)
Supplement: Supplementary file 1 — Student SBAR Assignment.docxSBAR-LA Rubric.docx [file mep_2374-8265.11184-s001.zip › B. SBAR-LA Rubric.docx]

APPENDIX B. SBAR-LA Rubric

| SBAR-LA Rubric  Final version of SBAR-LA: SBAR Brief Assessment Rubric for Learner Assessment is a scoring rubric developed for assessment of pre-professional students on interprofessional team training activities. | | | |
| --- | --- | --- | --- |
| SBAR Category | Sub-category | Description | Points^a^ |
| Situation | 1) Identifies self | Uses name for full credit | 0 or 1 |
|  | 2) Provides patient name | First, last, or both | 0 or 1 |
|  | 3) Provides a second patient identifier | i.e., DOB, age, gender, room number | 0 or 1 |
|  | 4) Expresses situation, issue, concern | To get full credit student needs to specifically state that "I need to speak to you", state "urgency," or "cannot wait" | 0 or 1 |
| Background | 1) States the context | “Are you aware of . . .” patient condition, reason for admission, and/or change in status | 0 or 1 |
|  | 2) States recent findings | i.e., mental status, lab values, vital signs, patient complaints | 0 or 1 |
|  | 3) Provides facts only | Does not provide unnecessary information | 0 or 1 |
| Assessment | 1) Provides summary assessment of problem | Need a summary of the underlying problem/concern which in this case is acute change in mental status and new information regarding coumadin | 0 or 1 |
| Recommendation | 1) Provides concrete suggested action | “Based on this assessment, I request/recommend that . . . i.e. come in and evaluate patient, get CT earlier.” For full credit also needs to include clear statement on confirmation of plan i.e. “Are you coming in, how do you want to be reached with new result.” | 0 or 1 |
|  | 2) Provides contact information | Phone number, texting app, pager | 0 or 1 |
| Total^b^ | | |  |
| Average | | |  |
| Global Effectiveness Rating (GER)^d^ | | Learner's ability to communicate elements of SBAR | 0,1,2 |
| ^a^SBAR points per subcategory: *unsuccessful* = 0; *successful* = 1.  ^b^Total points of 10 sub-categories (range 0 - 10).  ^c^Average score of subcategories = total score / n of sub-category scores (range 0 – 1)  ^d^GER points: *not effective* = 0; *somewhat effective* = 1; *very effective* = 2 | | | |
